# A Non-Destructive High-Speed Procedure to Obtain DNA Barcodes from Soft-Bodied Insect Samples with a Focus on the Dipteran Section of Schizophora

**DOI:** 10.3390/insects13080679

**Published:** 2022-07-27

**Authors:** Frederik Stein, Stefan Wagner, Nadine Bräsicke, Oliver Gailing, Carina C. M. Moura, Monika Götz

**Affiliations:** 1 Institute for Plant Protection in Horticulture and Forests, Julius Kühn Institute, 38014 Braunschweig, Germany; stefan.wagner@julius-kuehn.de (S.W.); nadine.braesicke@julius-kuehn.de (N.B.); monika.goetz@julius-kuehn.de (M.G.); 2 Büsgen Institute, Forest Genetics and Forest Tree Breeding, University of Göttingen, 37073 Göttingen, Germany; ogailin@gwdg.de (O.G.); carinamoura@uni-goettingen.de (C.C.M.M.)

**Keywords:** non-destructive DNA isolation, DNA barcoding, Diptera, soft-bodied arthropods, PCR inhibitor-resistant master mixes

## Abstract

**Simple Summary:**

Molecular genetic identification of insect species usually requires destructive DNA isolation for insects. Due to database gaps, errors, or low resolution of the examined gene region, a supplementary morphological identification may be necessary. To employ taxonomical experts efficiently, we developed a fast, economic, and simple non-destructive way for the molecular genetic species identification of flies and other soft-bodied insects.

**Abstract:**

While the need for biodiversity research is growing, paradoxically, global taxonomical expertise is decreasing as a result of the neglected funding for young academics in taxonomy. Non-destructive approaches for DNA barcoding are necessary for a more efficient use of this dwindling expertise to fill gaps, and identify incorrect entries in sequence databases like BOLD or GenBank. They are efficient because morphological re-examination of species vouchers is still possible post-DNA barcoding. Non-destructive approaches for Diptera with a comprehensive species representation or the consideration of diagnostic fragile morphological characters are missing. Additionally, most non-destructive approaches combine a time intensive and non-destructive digestion step with common DNA extraction methods, such as commercial kits or CTAB DNA isolation. We circumvented those approaches and combined a modified non-destructive TE buffer high-speed DNA extraction, with a PCR inhibitor-resistant PCR reaction system, to a non-destructive DNA barcoding procedure for fresh and frozen samples of the Schizophora (Diptera). This method avoids morphological impairment and the application of harmful chemicals, is cost and time effective, restricts the need for laboratory equipment to a minimum, and prevents cross-contamination risk during DNA isolation. Moreover, the study indicates that the presented non-destructive DNA barcoding procedure is transferable to other soft-bodied insects. We suggest that PCR inhibitor-resistant master mixes enable the development of new—and the modification of existing—non-destructive approaches with the avoidance of further DNA template cleaning.

## 1. Introduction

There is an increasing lack of taxonomical expertise globally due to the neglected funding of junior taxonomists [1,2]. DNA barcoding has become an adequate alternative to morphological identification [3]. Nevertheless, DNA barcoding is restricted by the gene region resolution, as reported by Meier et al. [4], for the cytochrome oxidase subunit 1 (COI) in dipteran species. Additionally, the identification via DNA barcoding can be constrained by sequence gaps in databases and incorrect database entries [5]. To augment databases and identify database errors, an integration of morphological identification of individuals and their respective barcode sequences is necessary. Commonly, destructive DNA extraction methods are required for small arthropods, which impedes later morphological species identification. Therefore, all samples of a study applying destructive DNA barcoding have to be morphologically identified before DNA barcoding to challenge database gaps and incorrect database entries. Simultaneously, the need for species identification is growing due to the increasing lack of taxonomical expertise, rising demand for biodiversity research in the context of insect decline [6], global warming [7], landscape fragmentation [8] or globalization [9], and new pests of cultivated plants [10] and their antagonist complexes.

An inefficient utilization of scarce taxonomical expertise will slow down and limit research requiring the morphological identification of arthropod species. This will negatively affect biodiversity research related to contemporary challenges. Therefore, non-destructive techniques for DNA-based species assignments are necessary to enable a morphological identification post-DNA barcoding, and to push forward the enhancement of sequence databases like BOLD (Barcode of Life database) or GenBank.

There are already non-destructive DNA isolation approaches developed for certain families [11,12,13], for specific purposes such as obtaining ancient DNA from museum samples [14,15], or developed for a broad range of arthropod orders [16,17,18,19,20]. Nevertheless, most previous studies about non-destructive DNA barcoding neglected the morphological particularities observed in Diptera, as they are often underrepresented, not present in studies, or their morphological preservation is not documented. Furthermore, non-destructive approaches for orders with a higher species representation are available. The combination of modified non-destructive digestion steps, followed up by a time-intensive CTAB-, kit-, or silica-based approach, is the main principle of most non-destructive methods for DNA isolation in arthropods among others [19,20,21]. For dipteran species, the non-destructive method proposed by Santos et al. [22] was evaluated for six species of Diptera. However, the sample size and sample history as well as questions regarding the conditions and length of preservation varied for different species, making the evaluation of this method challenging. Moreover, the approach included a digestion step that lead to a pigment loss, which can diffuse morphological characters, and was followed by a time-consuming CTAB DNA extraction. 

In this study, we propose a non-destructive high-speed approach applied to the cytochrome oxidase subunit 1 5′ region (COI-5P). We addressed the described issues related to Diptera without an impairment of morphological features, with a sufficient sample size, and a broad representation of the section Schizophora (Diptera). This method can be employed for DNA barcoding studies and enables preserving the voucher specimen’s morphology. We evaluated the method for different insect orders and it was most suitable for soft-bodied Diptera, in particular, Schizophora.

## 2. Materials and Methods

### 2.1. Sample Origin

The species samples of the insect orders Diptera (n = 38), Hymenoptera (n = 44), Lepidoptera (n = 23), and Coleoptera (n = 8) and the arachnid order Aranea (n = 5) came from laboratory and commercial rearing, insect pest monitoring by a forest protection service, and dry pitfall trap catches (Table 1). We conserved samples dry by freezing at a temperature < −20 °C in an upright freezer. 

To assess the impact of the non-destructive DNA extraction on arthropods’ morphology, we documented the morphological preservation for a selection of samples by means of focal plane pictures with a ZEISS Axiocam 506 color camera integrated in a binocular (Axio Zoom.V16), which were saved after optical sectioning as one image (Zen core v2.7, Zeiss). We took exemplary pictures of dry samples before DNA extraction and of the specimen in ethanol afterwards. For the tachinid species, *Exorista larvarum* (Linnaeus, 1758), we conducted photography (with the same equipment) of the diagnostic morphological characters, which are necessary to identify the species following the key of Tschorsnig and Herting [23]. In this case, we took pictures of untreated individuals and treated individuals in ethanol. We avoided taking pictures of dry untreated and in ethanol preserved treated samples, because at higher optical magnification the contrast of pictures differs between dry samples and samples preserved in ethanol.

### 2.2. DNA Extraction

The DNeasy^®^ Blood and Tissue Kit (Qiagen, Venlo, NL) (B&T Kit) was utilized to confirm our results from the non-destructive DNA barcoding procedure. We extracted DNA from *Lucilia sericata* and applied the DNA template as a positive amplification control. Furthermore, we tested the performance of primers for the COI-5P region for all species which were reared in laboratory (Table 1), with DNA templates obtained via the B&T Kit. Before the DNA extraction, which was carried out according to the manufacturer’s instructions, we homogenized individual specimens in the ATL buffer with stainless steel beads (diameter 1 × 5 mm, 2 × 2 mm and 3 × 1 mm) and at a frequency of 27 Hz for 2 min (Retsch Mill MM2, Retsch GmbH, Haan, Germany), to produce an optimal reaction surface.

**Table 1:** Overview of samples included in this study.

Order	Family	Species	Author of First Description	Origin	Institute	Stage	DNA extraction Modifications	Primer	No of Specimen				
									††	§§	¶¶	‡‡	†§
Araneae	Araneidae	*Mangora* species	O.P.-Cambridge, 1889	Meadow, dry pitfall trap	JKI GF †	Juvenile	Standard	C_LepFolF/CLepFolR [24]	0	0	0	5	5
Coleoptera	Curculionidae	*Otiorhynchus armadillo*	(Rossi, 1792)	Laboratory rearing	JKI GF †	Imago	Standard	C_LepFolF/CLepFolR [24]	0	0	0	5	5
Coleoptera	Curculionidae	*Pityogenes chalcographus*	(Linnaeus, 1761)	Laboratory rearing	JKI GF †	Imago	Standard	C_LepFolF/CLepFolR [24]	0	0	0	3	3
Diptera	Anthomyiidae	*Delia radicum*	(Linnaeus, 1758)	Laboratory rearing	JKI GF †	Imago	Standard	LCO1490/HCO2198 [25]	5	5	0	5	5
Diptera	Calliphoridae	*Lucilia sericata*	(Meigen, 1826)	Commercial rearing	JKI GF †	Imago	Standard	LCO1490/HCO2198 [25]	5	0	0	5	5
Diptera	Drosophilidae	*Drosophila hydei*	Sturtevant 1921	Commercial rearing	JKI GF †	Imago	Standard	LCO1490/HCO2198 [25]	10	0	0	10	10
Diptera	Tachinidae	*Blondelia inclusa*	(Hartig, 1838)	Diprionidae pupae	LFE §	Imago	Standard	LCO1490/HCO2198 [25]	5	0	0	5	5
Diptera	Tachinidae	*Exorista larvarum*	(Linnaeus, 1758)	Laboratory rearing	DISTAL	Imago	Standard	LCO1490/HCO2198 [25]	0	5	5	0	0
Diptera	Tachinidae	*Exorista larvarum*	(Linnaeus, 1758)	Laboratory rearing	DISTAL	Imago	Triton	LCO1490/HCO2198 25]	0	5	5	5	0
Diptera	Tachinidae	*Senometopia intermedia*	(Herting, 1960)	Ennominae cocoon	LFE §	Imago	Standard	LCO1490/HCO2198 [25]	1	0	0	1	1
Diptera	Tachinidae	*Senometopia pollinosa*	(Mesnil, 1941)	Ennominae cocoon	LFE §	Imago	Standard	LCO1490/HCO2198 [25]	1	0	0	1	1
Diptera	Tachinidae	species of Exorsitini tribe	NA	Diprionidae pupae	LFE §	Imago	Standard	LCO1490/HCO2198 [25]	1	0	0	1	1
Hymenoptera	Apidae	*Apis mellifera*	Linnaeus, 1758	Laboratory rearing	JKI BS ‡	Imago	Standard	C_LepFolF/CLepFolR [24]	0	0	0	5	5
Hymenoptera	Braconidae	*Diaeretiella rapae*	(M’Intosh, 1855)	Laboratory rearing	JKI GF †	Imago	Standard	C_LepFolF/CLepFolR [24]	0	0	0	5	5
Hymenoptera	Diprionidae	*Diprion* species	Schrank, 1802	Diprionidae pupae	LFE §	Imago	16 h	C_LepFolF/CLepFolR [24]	0	1	0	0	0
Hymenoptera	Diprionidae	*Gilpinia frutetorum*	(Fabricius, 1793)	Diprionidae pupae	LFE §	Imago	16 h	C_LepFolF/CLepFolR [24]	0	1	0	0	0
Hymenoptera	Diprionidae	*Gilpinia frutetorum*	(Fabricius, 1793)	Diprionidae pupae	LFE §	Imago	punctured + 16 h	C_LepFolF/CLepFolR [24]	0	1	0	0	0
Hymenoptera	Diprionidae	*Gilpinia variegata*	(Hartig, 1834)	Diprionidae pupae	LFE §	Imago	16 h	C_LepFolF/CLepFolR [24]	0	2	0	0	0
Hymenoptera	Diprionidae	*Gilpinia variegata*	(Hartig, 1834)	Diprionidae pupae	LFE §	Imago	punctured + 16 h	C_LepFolF/CLepFolR [24]	0	1	0	0	0
Hymenoptera	Diprionidae	unknown Diprionidae	NA	Diprionidae pupae	LFE §	Imago	16 h	C_LepFolF/CLepFolR [24]	0	1	0	0	0
Hymenoptera	Diprionidae	unknown Diprionidae	NA	Diprionidae pupae	LFE §	Imago	punctured	C_LepFolF/CLepFolR [24]	0	5	0	0	0
Hymenoptera	Diprionidae	unknown Diprionidae	NA	Diprionidae pupae	LFE §	Imago	punctured + 16 h	C_LepFolF/CLepFolR [24]	0	2	0	0	0
Hymenoptera	Diprionidae	unknown Diprionidae	NA	Diprionidae pupae	LFE §	Imago	Standard	C_LepFolF/CLepFolR [24]	0	5	0	5	5
Hymenoptera	Ichneumonidae	*Cratichneumon* species	Thomson, 1893	Ennominae cocoon	LFE §	Imago	Standard	C_LepFolF/CLepFolR [24]	0	0	0	1	1
Hymenoptera	Ichneumonidae	*Cratichneumon* species	Thomson, 1893	Ennominae cocoon	LFE §	Imago	Standard	C_LepFolF/CLepFolR [24]	0	1	0	1	1
Hymenoptera	Ichneumonidae	*Exenterus abruptorius*	(Thunberg, 1824)	Diprionidae pupae	LFE §	Imago	Standard	C_LepFolF/CLepFolR [24]	0	1	0	1	1
Hymenoptera	Ichneumonidae	*Exenterus* species	Hartig, 1837	Diprionidae pupae	LFE §	Imago	Standard	C_LepFolF/CLepFolR [24]	0	1	0	1	1
Hymenoptera	Ichneumonidae	*Heteropelma megarthrum*	(Ratzeburg, 1848)	Ennominae cocoon	LFE §	Imago	Standard	C_LepFolF/CLepFolR [24]	0	1	0	1	1
Hymenoptera	Ichneumonidae	*Hyposoter didymator*	(Thunberg, 1824)	Diprionidae pupae	LFE §	Imago	Standard	C_LepFolF/CLepFolR [24]	0	1	0	1	1
Hymenoptera	Ichneumonidae	*Lamachus frutetorum*	(Hartig, 1828)	Diprionidae pupae	LFE §	Imago	Standard	C_LepFolF/CLepFolR [24]	0	1	0	1	1
Hymenoptera	Ichneumonidae	*Pleolophus* species	Townes, 1962	Diprionidae pupae	LFE §	Imago	Standard	C_LepFolF/CLepFolR [24]	0	1	0	1	1
Hymenoptera	Ichneumonidae	species of Playbini Tribe	NA	Ennominae cocoon	LFE §	Imago	Standard	C_LepFolF/CLepFolR [24]	0	1	0	1	1
Hymenoptera	Ichneumonidae	unknown Ichneumonidae	NA	Diprionidae pupae	LFE §	Imago	Standard	C_LepFolF/CLepFolR [24]	0	3	0	3	3
Hymenoptera	Ichneumonidae	unknown Ichneumonidae	NA	Ennominae cocoon	LFE §	Imago	Standard	C_LepFolF/CLepFolR [24]	0	0	0	3	3
Lepidoptera	Erebidae	*Lymantria dispar*	(Linnaeus, 1758)	Laboratory rearing	JKI GF †	Imago	Standard	C_LepFolF/CLepFolR [24]	0	3	0	3	3
Lepidoptera	Geometridae	*Bupalus piniaria*	(Linnaeus, 1758)	Ennominae cocoon	LFE §	Imago	Standard	C_LepFolF/CLepFolR [24]	0	0	0	2	2
Lepidoptera	Geometridae	*Macaria liturata*	(Clerck, 1759)	Ennominae cocoon	LFE §	Imago	Standard	C_LepFolF/CLepFolR [24]	0	0	0	3	3
Lepidoptera	Noctuidae	*Mamestra brassicae*	(Linnaeus, 1758)	Laboratory rearing	JKI GF †	Imago	Standard	C_LepFolF/CLepFolR [24]	0	0	0	5	5
Lepidoptera	Erebidae	*Lymantria dispar*	(Linnaeus, 1758)	Laboratory rearing	JKI GF †	L3	Standard	C_LepFolF/CLepFolR [24]	0	1	0	1	0
Lepidoptera	Erebidae	*Lymantria dispar*	(Linnaeus, 1758)	Laboratory rearing	JKI GF †	L4	Standard	C_LepFolF/CLepFolR [24]	0	2	0	2	0
Lepidoptera	Erebidae	*Lymantria dispar*	(Linnaeus, 1758)	Laboratory rearing	JKI GF †	L5	Standard	C_LepFolF/CLepFolR [24]	0	2	0	2	0
Lepidoptera	Erebidae	*Lymantria dispar*	(Linnaeus, 1758)	Laboratory rearing	JKI GF †	L6	Standard	C_LepFolF/CLepFolR [24]	0	1	0	1	0
Lepidoptera	Erebidae	*Lymantria dispar*	(Linnaeus, 1758)	Laboratory rearing	JKI GF †	L7	Standard	C_LepFolF/CLepFolR [24]	0	4	0	4	0
									28	58	10	99	84

† Institute for Plant Protection in Horticulture and Forests, Julius Kühn Institute (Braunschweig, Germany), ‡ Institute for Bee Protection, Julius Kühn Institute (Braunschweig, Germany), § Brandenburg State Forestry Centre of Excellence (Eberswalde, Germany), ¶ Department of Agricultural and Food Sciences, University of Bologna (Bologna, Italy), †† samples included in selection of master mix, §§ samples included in evaluation of trouble shooting reaction system, ¶¶ samples included in development of DNA extraction with Triton x-100, ‡‡ samples included in evaluation of barcoding success rate in dependence to exoskeleton hardness and taxonomic units, †§ samples included in test for DNA concentration measurement suitability.

**Figure 1:**
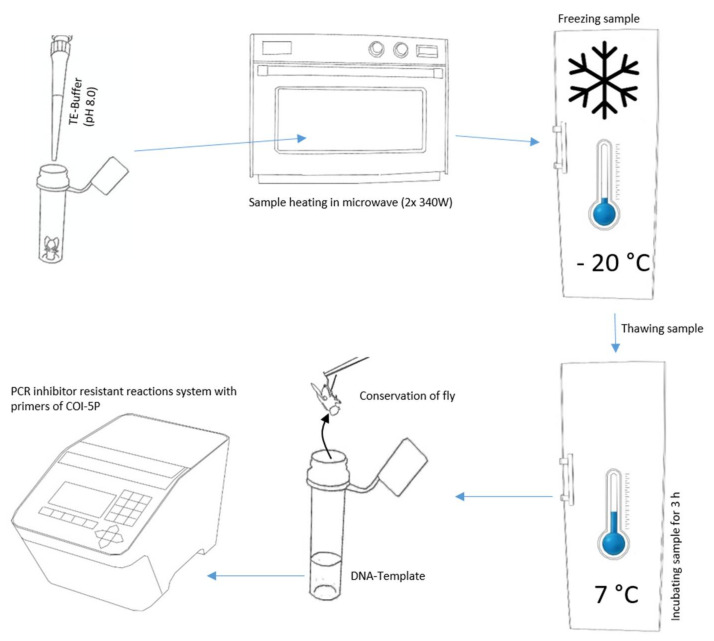
Essential steps for the non-destructive DNA barcoding procedure.

For the non-destructive DNA isolation, we modified the TE buffer extraction of Izumitsu et al. [26], as follows (Figure 1). First, we carefully placed individual specimens into tubes and vessels commonly applied in molecular genetics using forceps. In order to get the highest DNA concentration, it was desirable to have a minimum TE buffer volume to fully cover the body, and accordingly, different sizes of tubes and vessels analogous with insect size. We utilized vessels from 200 µL PCR reaction tubes to 10 mL tissue transportation tubes. By adapting the tube volume to body size, it was additionally possible to avoid samples floating to the top of the TE buffer in the case of air inclusions. Afterwards, we added TE buffer (10 mM Tris-HCL, 1 mM EDTA, pH 8.0) preheated to 30 °C, until complete coverage of the sample.

If the TE buffer was repelled by the specimen’s body surface, we decreased the surface tension of the TE buffer by adding the surfactant Triton X-100 [8.56 µmol/L]. Triton X-100 is only suspected to be a PCR inhibitor at high concentrations [27]. Subsequently, we moistened the flies by dipping them into modified TE buffer. Next, we removed the liquid from the sample with clean paper (household wipe), transferred it into a tube, and added TE buffer without Triton X-100. The remaining thin film of TE buffer with Triton X-100 on the fly surface enabled complete submersion of the sample.

With the following steps, we tried to generate micro cracks in the exoskeleton, cell membranes, or between chitin plates via alteration of cellular fluids’ density and aggregation state. First, we incubated the tubes in a microwave two times (Panasonic Pro II NE-1840, Panasonic, Osaka, JP) at a power of 340 W for one minute, with a break of 30 s between both heating periods. Second, the samples were frozen overnight at a temperature < −20 °C in an upright-freezer. Next, the samples were thawed and incubated at 7–8 °C for three hours in a fridge. Finally, we transferred the individuals with disinfected forceps to 70% ethanol and stored them at 7–8 °C for a later evaluation of their morphological preservation.

Beyond this, we tried to find possibilities for a troubleshooting of DNA isolation in case DNA concentrations from DNA templates obtained with the standard procedure were too low to accomplish DNA barcodes. We extended the diffusion time in the fridge to 16 h, punctured the arthropod samples to accelerate diffusion, and combined both treatments (Table 1).

We abstained from any further cleaning procedures and measured the DNA concentration of the TE buffer DNA templates obtained by the TE buffer DNA isolation, fluorometrically (Qubit^®^ dsDNA HS assay Kit, Thermo Scientific, Waltham, MA, USA), and initially photospectrometrically with the DS-11 FX Spectrometer/Fluorometer (DeNovix, Wilmington, DE, USA). Moreover, if the relationship between the measured DNA concentration and the result of sequencing remained unclear, we adapted further statistical assessment, as described in the stochastic analysis section.

### 2.3. PCR and Post PCR Treatment

We used primer systems to amplify the COI-5P region (Table 1). The region is suitable to determine species within the kingdom of Metazoa [3]. To ensure that primers or primer cocktails perform for all samples, we enquired on BOLD as to which primers or primer cocktails are most commonly applied to the broad taxonomical range of our specimens. Additionally, our selection relied on our own experience and on reports of Folmer et al. [25], Hebert et al. [3], and Gibson et al. [28] for Schizophora samples (Table 1). We confirmed our choice by applying the primers in a PCR with reference DNA templates obtained with the B&T kit.

Hence, we expected our DNA templates to contain PCR inhibitors. To avoid decreasing DNA concentrations, and risk of cross-contamination by time-consuming additional purification steps, we applied the DNA template to a PCR inhibitor-resistant reaction system (Figure 1). We tested four master mixes which, according to the manufacturers’ specifications, showed inhibitor resistance and proofreading function: UCP HiFidelity PCR master mix (UCP HiFidelity PCR Kit, Qiagen; abbr. UCP MM), AllIn^TM^ RPH master mix (highQu, Kraichtal, DE; abbr. AllIn MM), Phusion Flash High-Fidelity PCR master mix (Thermo Scientific; abbr. Phusion MM), and repliQa HiFi Though Mix^®^ (Quantabio, Beverly, USA]; abbr. repliQa MM). In addition, the MyFi^TM^ Mix (Bioline, London, UK]; abbr. MiFi MM), which has a proofreading function but no PCR inhibitor resistance, was applied as a control to test if PCR inhibitor resistance was necessary to amplify COI-5P sequences of DNA templates obtained by TE buffer DNA isolation.

We performed all PCR assays for the “standard reaction system” (SRS) in a total volume of 25 µL, using 2.5 µL DNA template, 0.5 µL of each primer [10 µM], 12.5 µL of the respective master mix, and 9.0 µL PCR grade water. We tested the “troubleshooting reaction system” (TSRS) in parallel to the SRS. This was done to guarantee the same DNA template quality for both reaction systems. The TSRS was developed for samples where the SRS did not lead to sequences with sufficient length and quality. For a TSRS, we modified the reaction system for the repliQa MM with an increased DNA concentration. We added 11.5 µL DNA template.

PCR programs followed the protocol PM7/129 (1) of the European and Mediterranean Plant Protection Organization [29] with an adjustment of the denaturation and annealing temperatures according to the user guidelines of the master mixes. Additionally, we adapted the cycle step times for the high-speed repliQa MM according to the user guide (Table 2). We conducted the PCR using a Biometra TOne Thermal Cycler (Analytic Jena GmbH, Jena, DE, Germany). To visualize the amplified fragments using standard agarose gel electrophoresis, we applied 10 µL of PCR products to 2% agarose gels with HDGreen^®^ Plus Safe DNA Dye (INTAS) as a DNA stain. For the gel documentation, we utilized the system Gel iX 20 (INTAS) imager.

Subsequently, we purified the PCR products with ExoSAP-IT^TM^ Express PCR Product Cleanup (Thermo Scientific). We deviated from the manufacturer’s instructions by diluting the ExoSAP-IT^TM^ Express reagent 1 to 10 with pure water. A feature of the ExoSAP-IT^TM^ Kit is that only a thermal cycler is needed to conduct the purification of PCR products. The cleaned up PCR products were sequenced in both directions by the Microsynth AG (Balgach, Switzerland). We assembled and edited the sequences with CLC Workbench 20.0.4 (Qiagen). To evaluate sequence quality and nucleotide reliability, Phred scores were used following the PM 7/129 (1) protocol of the European and Mediterranean Plant Protection Organization [29]. We will subsequently refer to the totality of steps leading to the COI-5P consensus sequence in a non-destructive manner as the “non-destructive DNA Barcoding procedure”.

Besides the preservation of morphology and the performance of DNA barcoding, we considered our approach from an economical point of view. To assess the TE buffer DNA extraction in the context of our work, we compared it to the widely-used B&T kit in entomology and further commercial DNA isolation or PCR product purification kits followed by digestion steps in the literature for non-destructive DNA isolation approaches. For the PCR, we compared costs of the master mix related to our approach with the best performance relative to other commonly used master mixes with a proofreading function. For the estimation, we took list prices for Germany in February 2022 without value added tax provided on manufactures’ or distributors’ homepages. Regarding time and effort, only the pure working time an experienced technical assistant needs to conduct the procedures of the B&T Kit protocol and non-destructive TE buffer DNA extraction was considered. Therefore, we excluded long waiting periods such as incubation in the digestion and TE buffers, or running the PCR in the thermal cycler, from calculations.

### 2.4. Stochastic Analyses and Assessment

For further statistical assessment, we first negatively tested our data sets for normal distribution by employing the “shapiro.test” function in R [30]. We used the Spearman’s correlation by employing the “cor.test” function with the method “pearson” [30] to evaluate if parameters are correlated. Besides the DNA concentration of the DNA template and the hardness of the exoskeleton, the success of DNA barcoding was a relevant parameter. We considered DNA barcoding as successful if we obtained a complete 658-bp-long COI-5P sequence, without ambiguous nucleotides, or if the sequences had the same taxonomic assignment in a BOLD query, like a complete sequence from the same species. Furthermore, we used this term to describe the performance of the presented method in the results. Hence, it is possible to fulfill the criteria for successful barcoding without a species identification due to a lack in BOLD. Species divergence thresholds were determined by the Refined Single Linkage (RESL) analysis employed by BOLD query. The algorithm considers that thresholds can vary between different taxa [31]. DNA barcodes were annotated as one, if successful, and as zero, if failed. If a complete COI-5P sequence of one species could not be obtained by the non-destructive DNA barcoding procedure, we used sequences from primer performance tests; in case no additional samples of a species were available, we downloaded complete sequences from BOLD. With the complete sequences, we conducted a database query to obtain reference results for incomplete sequences. We considered every sequence, which had ambiguous nucleotides or gaps for one or more bp, as incomplete.

We evaluated if the described methods of DNA concentration measurement are suitable for TE buffer DNA templates. We wanted to find out if it was possible to predict the success of DNA barcoding based on the measured DNA concentration. We tested if the DNA barcoding success rate positively correlated with the measured DNA concentration. If the concentration measurement was suitable, we would expect a significant correlation with a strong correlation rank coefficient. We selected data (n = 84) from DNA templates we applied to the SRS with the repliQa MM and no further modifications of the DNA extraction were applied.

Moreover, we assumed the permeability of the exoskeleton relative to the macromolecule DNA, which plays a key role for success of the described TE buffer DNA extraction. Besides the wax layer, the strength and rigidity of the integument resulting in the hardness of the exoskeleton are suspected to be relevant factors for permeability [32]. Therefore, we ranked every order, following the exoskeleton-hardness scale for bat prey by Freeman [33]. We deviated from the original scale by reassigning the third and fourth level, because none of the studied arthropods belonged to the third rank. The soft-bodied Diptera belonged to level one, the Lepidoptera were assigned to level two, the Hymenoptera matched to level three, and the Coleoptera were considered as the fourth level due to their hard exoskeleton. The caterpillars of *Lymantria dispar* and the juveniles of *Mangora* species were assigned to level one, because in the juvenile stages they are considered soft-bodied arthropods [34]. If the hardness of the integument affects the success of DNA barcoding, we would expect a significant negative correlation between the modified hardness scale of Freeman [33] and the success of DNA barcoding. To examine this assumption, we used data from samples (n = 99) that the SRS was applied to. For a second Spearman’s correlation, we excluded data (n = 79) from juveniles and caterpillars to take into account the fact that the juvenile stages can strongly deviate in their physiology and chemical composition compared with adult stages, besides the hardness of the exoskeleton [34]. For Lepidoptera, we were expecting the presented method would impair the morphological state by the loss of scales, which is the reason lepidopteran samples are never preserved in ethanol. However, we included them in the trials to have a reference for level two of the modified Freeman scale.

Additionally, we applied the Wilcoxon signed-rank test with “wilcox.test” function for paired samples [30] to test if different treatments of DNA templates caused a significant difference in results. 

To select the master mix with the best performance, we first tested all master mixes with DNA templates of *Lucilia sericata* obtained with TE buffer DNA extraction in a PCR. The subsequent selection was based on the results of a gel picture for five DNA templates. We excluded master mixes that did not perform for all DNA templates. For the remaining master mixes, we tested if the success rate for DNA barcoding per master mix significantly differed from each other by employing the Wilcoxon signed-rank test. We selected the data (n = 28) from all DNA templates of samples that were not impaired morphologically after simultaneous application of master mixes with the SRS.

To test if the TSRS generally increased the number of unambiguous nucleotides of edited DNA barcoding sequences in comparison to SRS, we selected data from DNA templates (n = 58) for which we conducted the SRS and TSRS, and sequences could be obtained for at least one reaction system. We utilized the Wilcoxon signed-rank test to confirm if significant differences were present in the number of unambiguous nucleotides between sequences based on the SRS and TSRS. Larger DNA template amounts bear the risk of a higher co-extracted PCR inhibitor concentration in the reaction system compared to smaller DNA template amounts. Therefore, we employed a second Wilcoxon signed-rank test (n = 42). We excluded sample data from the first data set, for which complete COI-5P sequences were obtained, by applying the SRS assuming that TSRS could deteriorate results for already-complete sequences accomplished via SRS. If the second test results in a significant increase of unambiguous nucleotides in sequences, and the first test was not found to be significant, this would indicate that an application of TSRS bears the risk of a deterioration of results by PCR inhibitors.

## 3. Results

### 3.1. Selection of Master Mixes

**Figure 2:**
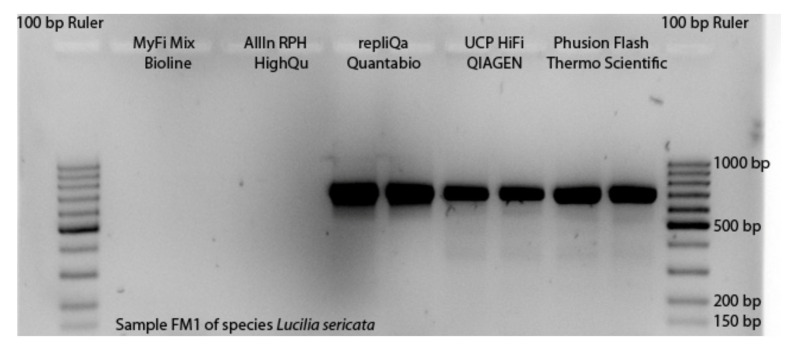
Performance of the five applied master mixes shown for one DNA template of *Lucilia sericata* amplified with primer pairs LCO1490/HCO2198.

To apply all the described modifications of TE buffer DNA extraction and PCR reaction system, and to assess the costs of the non-destructive DNA barcoding procedure, first the selection of the master mix with the best performance had to take place. The master mixes MyFi MM and AllIn MM only performed for one of five DNA templates of *Lucilia sericata* in the first trial. For four PCR products, no bands were visible on gel pictures, as Figure 2 illustrates for one sample. For the remaining three master mixes strong bands were visible for all samples (Figure S1). We continued the experiments with these master mixes displaying the best performance. For the DNA templates of *Drosophila hydei,* the performance of the three remaining master mixes was similar, showing successful amplification for nine out of ten DNA templates. However, we were not able to include the UCP MM in further trials, because the manufacturer was not able to provide it for several months.

The following experiments were executed with remaining master mixes repliQa and Phusion MM. With the repliQa MM, the barcoding was successful in 27 of 28 cases, including data of *Lucilia sericata* and *Drosophila hydei*. The Phusion MM did not perform for the DNA templates of tachinids hatched from pupae and the species *Delia radicum* (Linnaeus, 1758), so that 14 of 28 reaction systems did not lead to sequences. The Wilcox test considering 28 DNA templates showed the repliQa MM had a better performance (*p*-value < 0.001). Therefore, we conducted all the following trials with repliQa MM.

### 3.2. Success of Morphology Character Maintenance and DNA Barcoding

**Figure 3:**
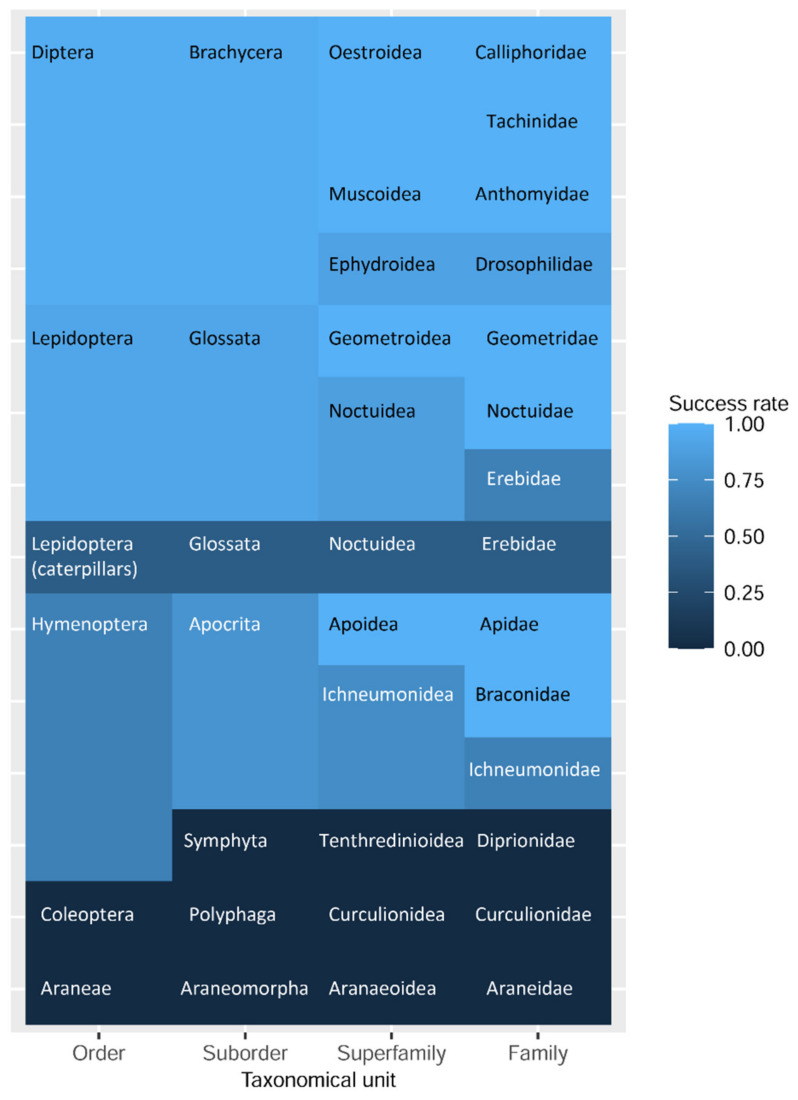
Heatmap of the non-destructive DNA barcoding success rate for examined taxa.

The maintenance of morphological state and the success of DNA barcoding varied between different examined taxonomical groups. The success rate of non-destructive barcoding ranged from 96.4% for flies, 92.3% for adult butterflies, and 53.3% for hymenopteran images, to 0% for beetles. For the caterpillars, the success rate was 30%, and for juvenile Araneae, the non-destructive DNA barcoding procedure did not perform (Figure 3).

### 3.3. Diptera (Brachycera: Schizophora)

**Figure 4:**
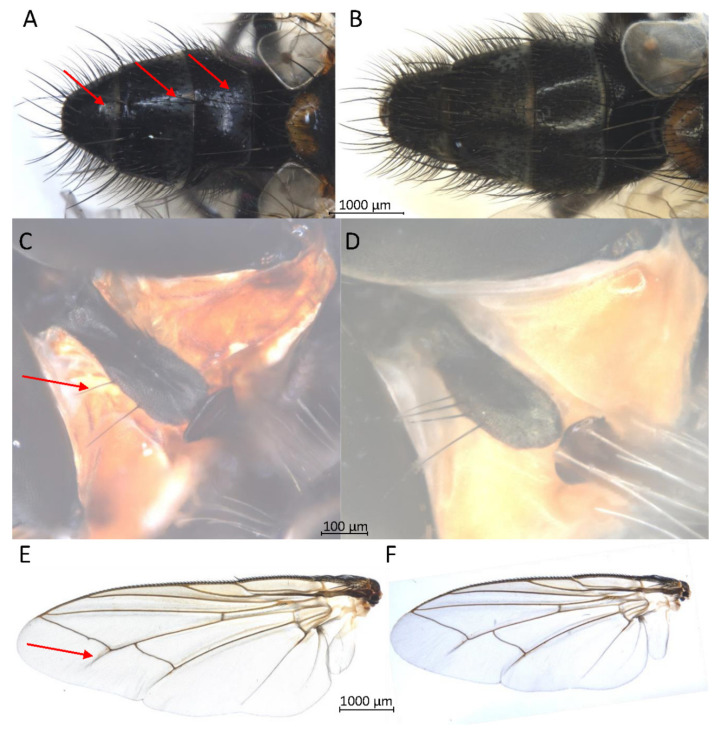
Three fragile diagnostic morphological characters of *Exorista larvarum* tagged by red arrows; all pictures of samples were taken in ethanol. (**A**) abdomen with dusting without treatment. (**B**) abdomen after DNA barcoding. (**C**) prosternum with hairs at the side edge without treatment. (**D**) prosternum after DNA barcoding. (**E**) wing with shadow fold without treatment. (**F**) wing after DNA barcoding.

For Brachycera (Schizophora), morphological impairment due to the TE buffer extraction for all individuals was avoided. No loss of bristles or pigments, and no impairments of wing membrane stability were observed (Figure S2). For *Exorista larvarum,* with standard TE buffer DNA extraction, we observed some damages of dusting in areas that were not wetted by TE buffer. With the submersion of samples in the TE buffer amended with Triton X-100, we were able to avoid a repulsion of the TE buffer and consequently, the damage of dusting. Besides others, the fragile diagnostic morphological characters such as hairs on the side edge of the prosternum and shadow folds in the wing membrane of *E. larvarum* did not show any impairment by TE buffer DNA extraction (Figure 4). Furthermore, the cerci remained extendable. For the 33 samples of the brachyceran section Schizophora, including eight species representing four families, a successful DNA barcoding with repliQa MM was performed in 32 cases.

### 3.4. Hymenoptera (Symphyta, Apocrita)

**Figure 5:**
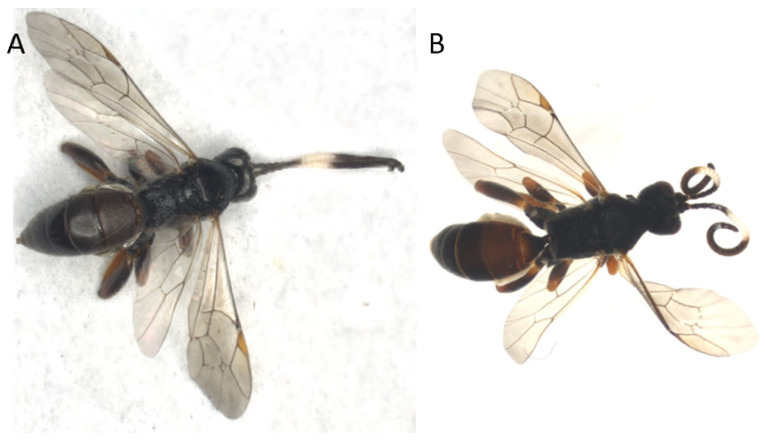
Curling of antenna by TE buffer DNA extraction for an Ichneumonidae sample. (**A**) before treatment, picture taken in a dry setting. (**B**) after TE buffer DNA extraction, picture taken in ethanol.

For Hymenoptera, the morphological state before and after treatment was similar to Schizophora (Figure S3), with the exception of the antenna of Ichneumonidae individuals, which curled in some cases (Figure 5). Furthermore, a longer incubation time in the TE buffer had no visible effect on the samples.

The non-destructive DNA barcoding procedure showed different DNA barcoding success rates for different groups of Hymenoptera (Figure 3). For all *Apis mellifera* (Apidae) samples, we obtained two complete sequences, and three incomplete sequences, which were sufficient to conduct a successful DNA barcoding. The results for Ichneumonidea were inconsistent. For five Braconidae individuals of the species *Diaeretiella rapae*, two incomplete and three complete sequences were obtained. The incomplete sequences were sufficient to obtain the same results as a complete sequence, hence it was estimated as a successful DNA barcoding. For the family Ichneumonidae, we obtained four complete sequences, two incomplete sequences, and in eight cases, no sequence. For 11 samples, one sequence was degraded by TSRS and five sequencing results were identical to the results based on SRS. In addition, for five samples with missing amplification in SRS, three complete sequences were obtained. Furthermore, we obtained one incomplete sequence enabling a successful DNA barcoding and one incomplete sequence, which did not allow a successful DNA barcoding.

For five individuals of the Diprionidae, for both the SRS and TSRS, DNA barcoding was not successful. One short sequence was obtained for SRS; for the remaining samples no sequences could be obtained. Therefore, we applied further modifications for DNA isolation. For five individuals, which remained in the fridge for 16 h after the thawing step, we obtained three short incomplete sequences which were not suitable to obtain results via database queries, and for two samples no results were accomplished. An additional combination with TSRS enhanced the results in four out of five cases. We received three complete sequences, one incomplete sequence, and in one case, no sequence could be received. The shorter fragment was not sufficient to identify the individual to the species level, nevertheless the genus identification was successful. The five punctured samples gave one short fragment of low quality. The TSRS did not improve the result. The combination of both modifications for the SRS resulted in one complete sequence and one incomplete sequence. In three cases, no result could be obtained. The TSRS could improve the shorter sequence to a complete sequence. Therefore, for two samples, the DNA barcoding was successfully conducted.

### 3.5. Further Samples (Insects: Coleoptera, Lepidoptera, Arachnida: Araneae)

Our method had restrictions for the remaining arthropod groups because of morphological impairments or an unreliable DNA barcoding success rate. As we expected for adult lepidopteran samples, the morphological state for all individuals was impaired due to a loss of wing scales during TE buffer extraction (Figure S4). The documentation of morphology for Coleoptera, Araneae, and lepidopteran caterpillars was not conducted, because it was redundant due to the low success rates of DNA barcoding. For the Coleoptera and juvenile spiders, gel pictures only showed weak bands. For these samples, we did not obtain sequences. For the different caterpillar stages of *Lymantria dispar,* we obtained two incomplete sequences and one complete sequence allowing a successful database query out of ten samples. Nevertheless, for adult Lepidoptera samples of four species (Erebidae, Geometridae, Noctuidae) the DNA barcoding succeeded 12 out of 13 times. The fail belonged to the species *Lymantria dispar*, where for two of three cases DNA barcoding was successful, accomplished with one complete and one incomplete sequence. For the third *Lymantria dispar* sample, we did not obtain any sequence with SRS. With the TSRS we were able to enhance the sequence to an incomplete sequence, which was sufficient to assign the sequence to the genus.

**Figure 6:**
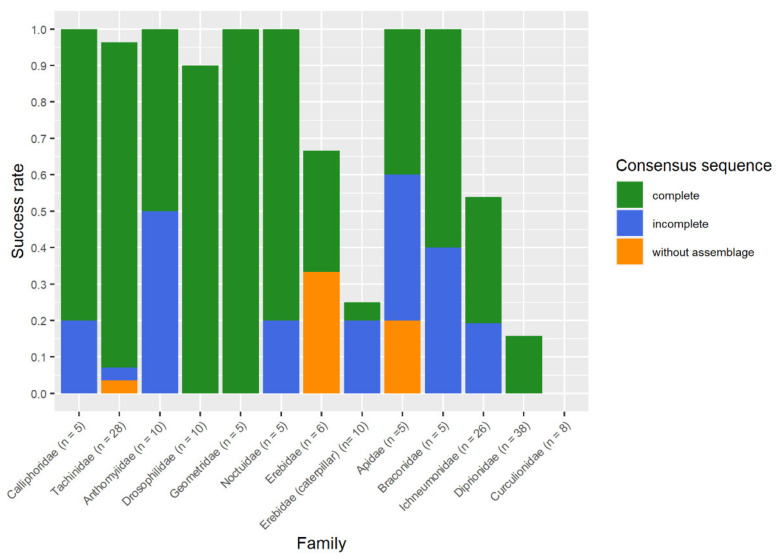
Consensus sequences state enabled a successful non-destructive DNA barcoding obtained with the repliQa HiFi Though Mix^®^.

For all 176 reactions (Table S1) conducted with the repliQa MM during the study, we obtained 75 complete sequences, 38 incomplete sequences, and in 63 cases, no sequences were obtained. In 13 cases, incomplete sequences were due to non-overlapping forward and reverse sequences. In five cases we did not obtain the forward sequence, and in eight cases the reverse sequence was not available. Twice, both sequences were available but too short for an assemblage. A successful DNA barcoding mostly was obtained with complete sequences and incomplete sequences. In some cases, consensus sequences based on a forward or reverse sequence were sufficient for a successful DNA barcoding (Figure 6).

### 3.6. Statistical Analysis

We found that photospectrometric and fluorometric DNA concentration measurements were not suitable to predict the success of DNA barcoding for DNA templates obtained by TE buffer extraction. We had to exclude photospectrometric measurements because they did not give plausible results. For the fluorometric measurements, we did not find a significant correlation between the success of DNA barcoding and measured DNA concentration, based on 84 DNA templates. We have to mention that sometimes DNA templates obtained by TE buffer extraction show a light to conspicuous colouration. We measured a concentration of 6.07 ng/µL for the DNA template of sample D.h.18 and we were not able obtain a sequence, while we measured a DNA concentration of 0.02 ng/µL for the DNA template of sample KF10 and we could amplify the complete 658-bp-long sequence, allowing a species identification. This fact illustrates the low correlation rank coefficient of ρ = 0.09375.

The “cor.test” between the DNA barcoding success rate and the modified Freeman scale showed a significant negative correlation (*p* < 0.001) with a correlation rank coefficient of ρ = −0.577, if only data of adult life stages were considered. If juveniles and larvae were included, a significant negative correlation remained (*p* < 0.001) with a weaker correlation rank coefficient (ρ = −0.24) (Figure S5).

For all the samples where we conducted the SRS and TSRS, we could not find a significant difference in unambiguous nucleotide number due to the reaction system. In 19 cases, the number of unambiguous nucleotides could be increased, while for 30 cases there were no changes in the results, and in nine cases the TSRS decreased the number of unambiguous nucleotides. For the samples in which SRS did not result in complete COI-5P sequences, the TSRS significantly increased the number of unambiguous nucleotides (*p*-value = 0.009). In 16 cases, the number of unambiguous nucleotides was raised, another 16 results did not change, and the number of unambiguous nucleotides was reduced five times.

The economic considerations for the method presented in this paper show the procedure produces lower material costs and takes less pure working time compared with approaches where digestion steps and commercial kits are combined. The material costs for the TE buffer and consequently for DNA extraction are negligible (0.0004 € for 1 mL). In contrast DNA extraction with the B&T kit costs 2.80 € per sample. Other commercial kits applied in the context of post-digestion steps for non-destructive DNA isolation approaches are the NucleoSpin Tissue kit from Machery Nagel [35] at 2.64 € per sample, and the QIAquick PCR Purification Kit from Qiagen [36] at 1.99 € per sample.

Costs for PCR master mixes with proof-reading polymerases of different manufacturers can range from 0.22 € for HOT FIREPol Blend master mix (Solis BioDye, Tartu, Estonia), over 0.65 € for MyFi MM, to 1.73 € for UltraRun LongRange PCR master mix (Ultra LongRange PCR Kit, Qiagen).

Based on this price information, the total material cost for a non-destructive approach including commercial DNA isolation or PCR product purification kits could vary between 2.21 € and 4.53 € for one sample. The total costs for the non-destructive DNA barcoding procedure, including the DNA isolation and PCR steps, amounts to 2.24 € for one sample.

The work effort for production of TE buffer is negligible, as multiple litres can be prepared in once. The pure working time for the TE buffer extraction for 20 samples is 30–45 min. In contrast, it takes 2 h for DNA isolation with the B&T kit. The work effort for the PCR is identical.

## 4. Discussion

Despite previous studies on non-destructive DNA isolation approaches, including dipteran samples, studies considering morphological integrity, sample size, consistent sample history, or a comprehensive representation of the order by sufficient species in an adequate manner are missing [11,12,15,16,18,19,22]. This issue might be caused by a focus on other taxa or lower taxonomic ranks within Diptera or a lack of available species. With our approach, we address this lack of studies for the dipteran section of Schizophora. The non-destructive DNA barcoding procedure should have transfer potential to the entire dipteran order depending on the exoskeleton hardness.

We were able to circumvent typical morphological issues associated with non-destructive DNA extraction approaches of several studies. For non-destructive approaches often a loss of pigments is described [19,22] or visible in pictures [11]. Another special issue is the stability of wing membranes. These are often damaged, while other arthropod orders do not show impairments [11,16,19]. In some studies the remaining morphologically relevant “diagnostic characters” are considered [12,37], enabling the morphological identification of the examined group. Nonetheless, it is not mentioned if other morphological characters became impaired, which could be relevant for the morphological identification of further arthropod groups. Ritter et al. [18] did not report on the morphological preservation of the analyzed samples. We assume that methods with longer heating steps, also including the above-mentioned methods, could affect the condensation of proteins, and thus impede the extendibility of the cerci, a pivotal property for species identification of several tachinid species [23]. Longer heating steps are present in all approaches with digestion steps for non-destructive DNA isolation [11,15,18,19,21,22,35,36,38,39,40,41,42,43,44,45,46,47,48,49]. Longer or repetitive heating steps are also used in direct PCR methods [13,37,41], the HotSHOT method [20,41], the Chelex method [35,43], the TE-boiling method [50], and the TNES method [51] for arthropods.

To avoid this issue, it is necessary to extend the cerci before DNA extraction. Nevertheless, with our proposed non-destructive method, the extension of the cerci can be performed after the DNA extraction, in cases where morphological identification is necessary. Additionally, in our own experience, working with digestion steps bears the risk of loosening of bristles, loss of pigments, and impairment of wing stability. A general disadvantage of methods including digestion steps is the need for longer washing procedures, which are time consuming.

The sonication method by Hunter et al. [52] avoids any sample heating and is an interesting high-speed approach. However, the authors report that the success rate of the method to obtain barcode sequences was only 66% for one dipteran species, which is lower compared to our success rate of 96.4%. They assumed low reliability was due to PCR inhibitors or a too low DNA concentration. Stamper et al. [53] validated the sonication method and found on average a lower Phred score and shorter barcode sequences compared with barcode sequences based on DNA templates isolated with the B&T Kit. Unfortunately, Stamper et al. [53] did not mention if they applied a PCR inhibitor-resistant PCR reaction system. However, we suspect a combination of the sonication method with the repliQa MM could be rewarding. Similarly, direct PCR approaches could be enhanced, which can also be restricted by PCR inhibitors depending on taxa and tissue [37]. Additionally, direct PCRs often are semi-destructive, because only a leg is given to the reaction to avoid high concentrations of co-extracted PCR inhibitors. This could be circumvented with the utilization of PCR inhibitor-resistant repliQa MM and enable a non-destructive direct PCR for a broader range of small arthropods.

In contrast to previous studies for our non-destructive DNA barcoding procedure, we did not detect any morphological impairment related to different species out of several schizophoran families. Additionally, we confirmed the method by the documentation of intact fragile diagnostic morphological characters of *Exorista larvarum* post non-destructive DNA procedures. The pivotal requirement for the determination of several tachinid species is the preservation of the extendibility of the cerci.

Besides morphological impairment, a comprehensive experimental design with dipteran samples is missing in most other studies. We found two studies considering Diptera, in which no morphological impairments were found. However, in these studies the Diptera were restricted to one or two species [49] or had a semi-destructive character [37]. The underrepresentation of Diptera can be found in several studies [11,16,19,20,37]. In some papers the sample sizes are not mentioned or remain unclear [17,20,40]. Furthermore, the history of the samples, whether they were fresh or frozen, and whether they were preserved in ethanol or spent years as pinned dry samples is not addressed [17,20,35,39,43,48]. Santos et al. [22] considered five species out of four dipteran families; nonetheless, samples sizes per family and sample history were not consistent. Giantsis et al. [40] adapted their method to four species of Diptera. However, samples sizes per species were unknown, three species were represented by frozen samples, and one species was represented by in ethanol-preserved samples. The consistency of sample sizes and history is important to evaluate strengths, weaknesses, and adaptation potential of non-destructive approaches.

For our study, the access to laboratory rearings of several insect pests and insect pests’ antagonists allowed us to cover four of five levels of the Freeman scale. Therefore, we were able to avoid small sample sizes per species or diverse sample histories and underrepresentation of the examined taxon, ensuring a broad range of several families in most cases. This enabled us to address issues that otherwise had not been recognized. The Phusion MM performed well in the first trials for the species *Drosophila hydei* and *Lucilia sericata*, while we did not obtain COI-5P sequences from tachinid species and the Anthomyiidae *Delia radicum*. Species like *Exorista larvarum* had surface properties, which repelled the TE buffer. Therefore, we had to adapt our method with the Triton step.

Furthermore, economic considerations demonstrated that the non-destructive DNA barcoding procedure is less cost intensive than kit-based methods. An exception could be a combination of the HOT FIREPol Blend master mix with the QIAquick PCR Purification Kit from Qiagen, employed by Nagarajan et al. [36]. From our own experience, the HOT FIREPol Blend master mix performs only with DNA templates with higher DNA concentrations compared with the MiFi^TM^ master mix, hence a possible application strongly depends on the DNA concentration of the DNA template isolated with a non-destructive approach.

Moreover, our method is less time consuming than other methods, requiring no digestion steps and elaborate washing steps afterwards. Additionally, those washing steps bear the risk of cross contaminations and often include the application of harmful chemicals.

Furthermore, a special feature of our method is that there is no limitation for larger dimensions of arthropod bodies, because the tube or vessel size can be adapted. In contrast, for tiny insects, the possible amount of DNA template that can be applied to the PCR is restricted, because less than 2.5 µL TE buffer is necessary to cover the entire insect. Hence, the DNA concentration in the final reaction volume is too low. In those cases, direct PCRs [13,37] could be a useful complement to our method.

Moreover, our results point out further limitations of the non-destructive DNA barcoding procedure. We suggest the exoskeleton hardness represented by the modified Freeman scale plays a key role in restricting the success of DNA barcoding. The strong negative correlation between the modified Freeman scale and the success rate of non-destructive DNA barcoding procedure, excluding juvenile stages, indicates hardness is related to the permeability of the exoskeleton. As suggested by Klowden [34], for the permeability, different layers in the structure of the exoskeleton are pivotal.

The wax layer plays a key role in permeability via the transpiration rate of water from insect samples [54]. The wax layer should inhibit diffusion of DNA due to the size of the macromolecule. The chemical properties like hardness, melting point, and impact strength in relation to temperature depend on the ratio of hydrocarbons and long chain alcohols, which varies between different taxonomical groups [34]. Consequently, the steps of heating and freezing during the TE buffer extraction could have an impact on the wax layer and enable a better permeability of DNA and other chemical components. This impact could be limited by the cement layer, which covers the wax layer. However, this cement layer is not always present. For example, for honeybees (*Apis mellifera*), which were also included in the trials, it is missing [34].

Further factors are the rigidity and thickness of the whole exoskeleton, which have a strong influence on the hardness [32], and should be indirectly represented by the modified Freeman scale. The “cor.test” of the modified Freeman scale and the success of the non-destructive DNA barcoding procedure shows the limitation of the presented method due to the hardness of the exoskeleton. The thickness of the exoskeleton varies in different orders due to the thickest layer, the endocuticle. The endocuticle can have a strength between 10 and 200 µm. Soft-bodied arthropods are characterized by a reduced endocuticle [34]. The thickness could mechanically decrease the probability of DNA molecules passing through the integument.

Besides further physiological properties of the integument including ecdysial lines, pore canals, or punctual hardened body parts such as mandibles or drilling ovipositors, which can have a local influence on the permeability [34], co-extracted PCR inhibitors could play a limiting role for the presented method. We suspect in these cases, the PCR inhibitor concentration the master mix resists was exceeded. The rigidity depends on the cuticular proteins. Higher concentrations of cuticular proteins could have contributed to high co-extracted PCR inhibitor concentrations, exceeding PCR inhibitor resistance of the reaction system. Nevertheless, the negative correlation between the DNA barcoding success rate and the Freeman scale (ρ = −0.24), including the data of juvenile stages, does not fully explain the limitations of this method as a result of exoskeleton hardness. After the data exclusion of juvenile samples, the significance and correlation rank coefficient enhanced, which indicates that there are further limiting factors related to the properties of the mentioned samples beyond the hardness of the endocuticle. In the case of caterpillars, high protein content [55] could have substantially contributed to the exceeding of the master mix PCR inhibitor resistance. Similar PCR inhibiting compounds of the *Mangora* samples (Araneae), such as the silk protein [56], could be related to exceeding PCR inhibitor resistance of the applied master mix.

Moreover, we suspect higher PCR inhibitor concentrations in the TSRS, compared with SRS, to be a major factor for the TSRS to not perform significantly better than the SRS. Nevertheless, the TSRS could significantly enhance results for DNA templates where the SRS did result in incomplete or no COI-5P sequences. Therefore, we recommend using SRS routinely and applying the TSRS only in case the SRS does not perform optimally.

Additional, the results of photospectrometric and fluorometric measurements of DNA concentration were not suitable to predict if SRS will result in successful DNA barcoding. We assume co-extracted substances, in parts visible in DNA template colouration probably falsified wavelength measurements or influenced signal strength. Furthermore, high PCR inhibitor concentrations could have made DNA concentration measurements unsuitable in terms of predicting results, by inhibiting PCRs with high DNA concentrations.

Besides the described limitations, the results for DNA extraction modifications for Diprionidae indicate that there could be a potential to augment DNA isolation for groups that are limited to reliably perform a non-destructive DNA barcoding procedure, due to the hardness of their integument. The modifications of DNA isolation indicate longer incubation in TE buffer and puncturing could enhance the DNA concentration of DNA templates. Nevertheless, the DNA concentration was still low and the non-destructive DNA barcoding procedure with TSRS did not perform for the Diprionidae in a reliable manner. In contrast to the standard procedure of DNA extraction, the DNA templates of samples that were incubated in TE buffer for a longer time, were punctured, or were incubated for a longer time and were punctured, resulted in COI-5P sequences in some cases. To be able to apply augmented diffusion time as a troubleshooting modification, a small amount of DNA template could be taken after 3 h and be used for the standard procedure. In case the PCR is not successful, a repetition of PCR after 16 h of diffusion time can be conducted. The results are not directly comparable between different modifications, because we utilized different individuals of different species from one family. Hence, we encourage researchers to apply longer diffusion times for samples of the modified Freeman scale three. Therefore, for the Ichneumonidae, augmenting the diffusion time could have been an adequate step to adapt the non-destructive DNA barcoding procedure to reach reliable results for all samples and perhaps without applying the TSRS.

We assume our method is adaptable to a broader range of soft-bodied arthropods. A transfer to other Diptera seems plausible. In consideration of Aoyama et al. [50], who developed a similar method called “TE-boiling method” for Collembola with a common master mix, our protocol should be transferable to Collembola. Furthermore, we conclude most groups that belong as adults to the modified Freeman scale one or two, and in some cases three, can be identified via DNA barcoding with our approach. In addition, an extension of diffusion time could lead to an improvement of the non-destructive DNA barcoding procedure in cases of too low DNA concentrations. Nevertheless, there are limitations, including the unreliable DNA barcoding success demonstrated for Diprionidae, Coleoptera, juvenile Araneae, and larval lepidopterans. Moreover, the loss of lepidopteran wing scales and the curling of ichneumon antenna demonstrated that the non-destructive DNA barcoding procedure will not always lead to a complete maintenance of morphological characteristics. Hence, we recommend that comprehensive pre-tests for a possible transfer to other orders are necessary, especially for orders of level three of the modified Freeman scale.

In spite of the restriction of investigations to fresh or frozen samples, we see a broad range of applications for the presented method. It is optimally suited for schizophoran flies. Conceivable applications include the examination of catch samples from landing or beating nets and catches with live traps, without entrapment liquid. Furthermore, parasitoid complexes can be revealed by hatched parasitoids from host pupae of interest, and the fast identification of new and immigrated insect pests is possible.

Prospectively, we think PCR inhibitor-resistant master mixes could be beneficial for samples caught by traps equipped with entrapment liquids. Especially for entrapment liquids, such as saturated solutions of benzoic acid [57], with properties like low evaporation rate, enabling longer time periods in the field is suspected to inhibit PCRs [58]. Beyond this, an application of the presented method to trap samples could be rewarding.

## 5. Conclusions

The published methods for non-destructive DNA isolation, including Diptera, show a lack of sufficient sample size, consistent sample history, representing species, or morphological integrity. Considering these facts, we developed a non-destructive DNA barcoding procedure for fresh and frozen adult samples of the Schizophora, a section of Diptera. The method is economical, time efficient, and avoids cross contaminations and the application of harmful chemicals. The laboratory equipment is constrained to a thermal cycler, microwave, fridge, and freezer. The presented method has a transfer potential to further arthropod groups of the modified Freeman scale levels one, two, and with restrictions, to level three. PCR inhibitor-resistant master mixes could enable a new generation of non-destructive high-speed PCR-based approaches—and enhance existing high-speed PCR-based approaches—by avoiding laborious and cost intensive washing of PCR inhibitor steps.

## Figures and Tables

**Table 2:** Cycling conditions following EPPO Bulletin [29] adapted to the master mixes’ manuals.

Thermal Cycler Step	repliQa MM	UCP & AllIn MM	Phusion & MyFi MM	No of Cycles	Go To
Initial Denaturation	98 °C (1 min)	95 °C (3 min)	98 °C (3 min)	1	
Denaturation	98 °C (10 s)	95 °C (30 s)	98 °C (30 s)	5	
Annealing	45 °C (5 s)	45 °C (30 s)	45 °C (30 s)		Step 2
Elongation	68 °C (2 s)	72 °C (1 min)	72 °C (1 min)		
Denaturation	98° C (10 s)	95° C (30 s)	98° C (30 s)	35	
Annealing	51 °C (5 s)	51 °C (1 min)	51 °C (1 min)		Step 5
Elongation	68 °C (2 s)	72 °C (1 min)	72 °C (1 min)		
Extension	-	72 °C (10 min)	72 °C (10 min)	1	

## Data Availability

For every sample we received COI-5P sequences we uploaded the best consensus sequences to Barcode of Life Database. You can find the genetic and meta data, when you search for the NODES project on the BOLD homepage.

## Supplementary Materials

The following supporting information can be downloaded at: https://www.mdpi.com/article/10.3390/insects13080679/s1, Figure S1: Performance of the five applied mastermixes shown for one DNA template of *Lucilia sericata* amplified with primer pairs LCO1490/HCO2198.; Figure S2: *Delia radicum* sample. A, before treatment, picture taken in a dry setting. B, after TE buffer DNA extraction, picture taken in ethanol.; Figure S3: *Diaeretiella rapae* sample. A, before treatment, picture taken in a dry setting. B, after TE buffer DNA extraction, picture taken in ethanol.; Figure S4: *Mamestra brassicae* sample. A, before treatment, picture taken in a dry setting. B, after TE buffer DNA extraction, picture taken in ethanol.; Figure S5: Correlation between modified Freeman scale and non−destructive DNA barcoding success rate with and without juvenile.; Table S1: Data sheet for all PCR reactions.
