# Supplementary figures and images for: A Non-Destructive High-Speed Procedure to Obtain DNA Barcodes from Soft-Bodied Insect Samples with a Focus on the Dipteran Section of Schizophora
Source: Insects. 2022 Jul 27;13(8):679. doi: 10.3390/insects13080679 (PMC9409269; doi:10.3390/insects13080679)

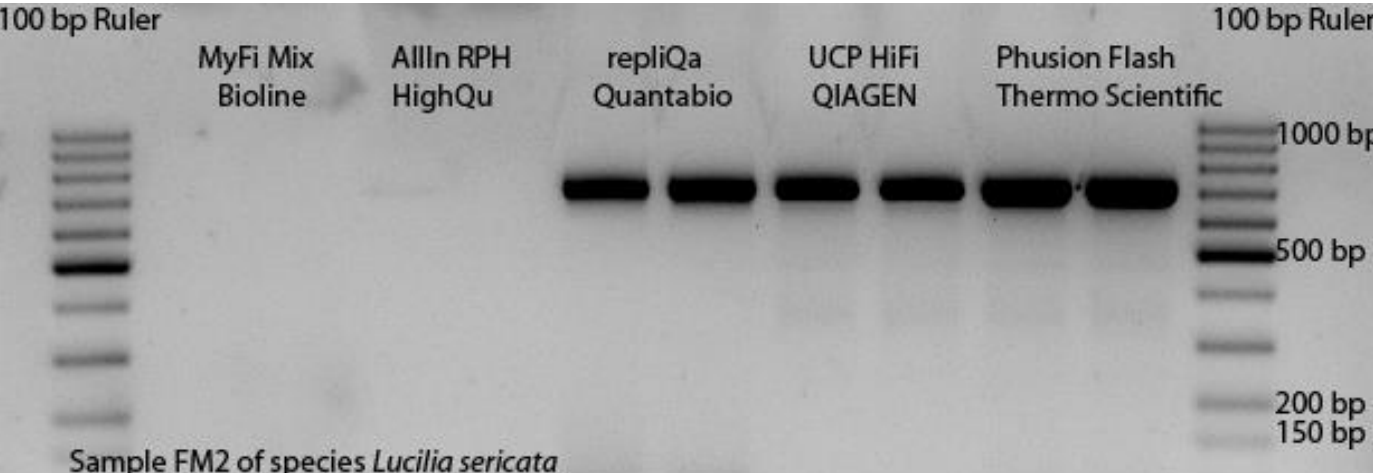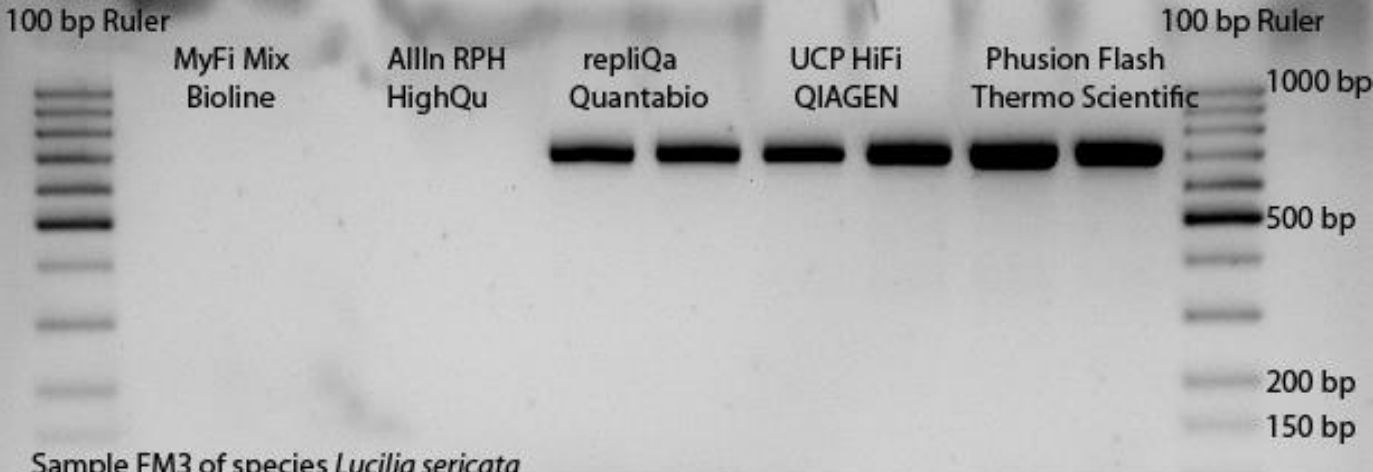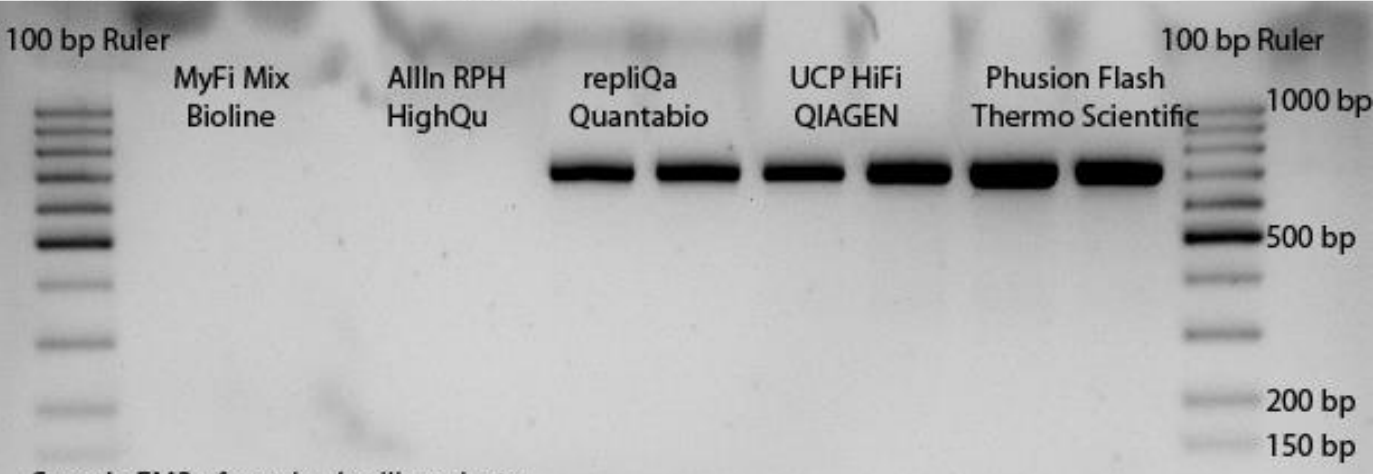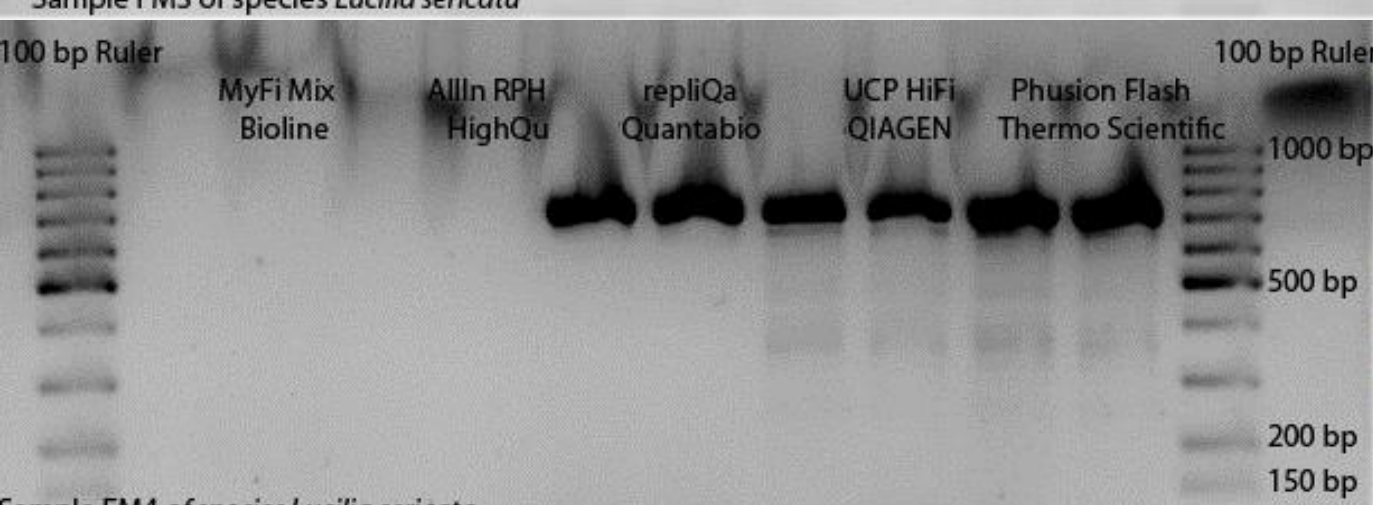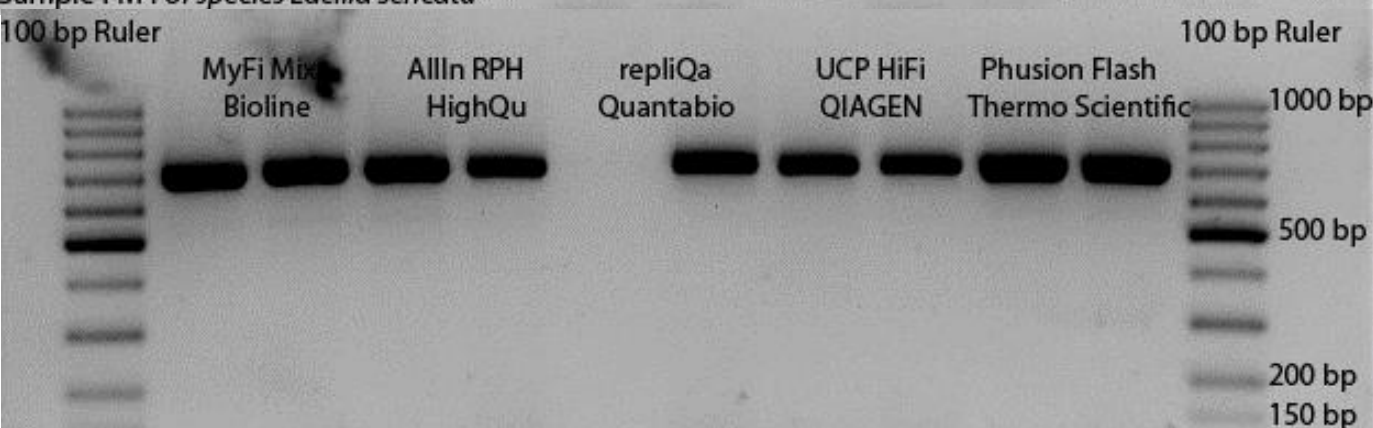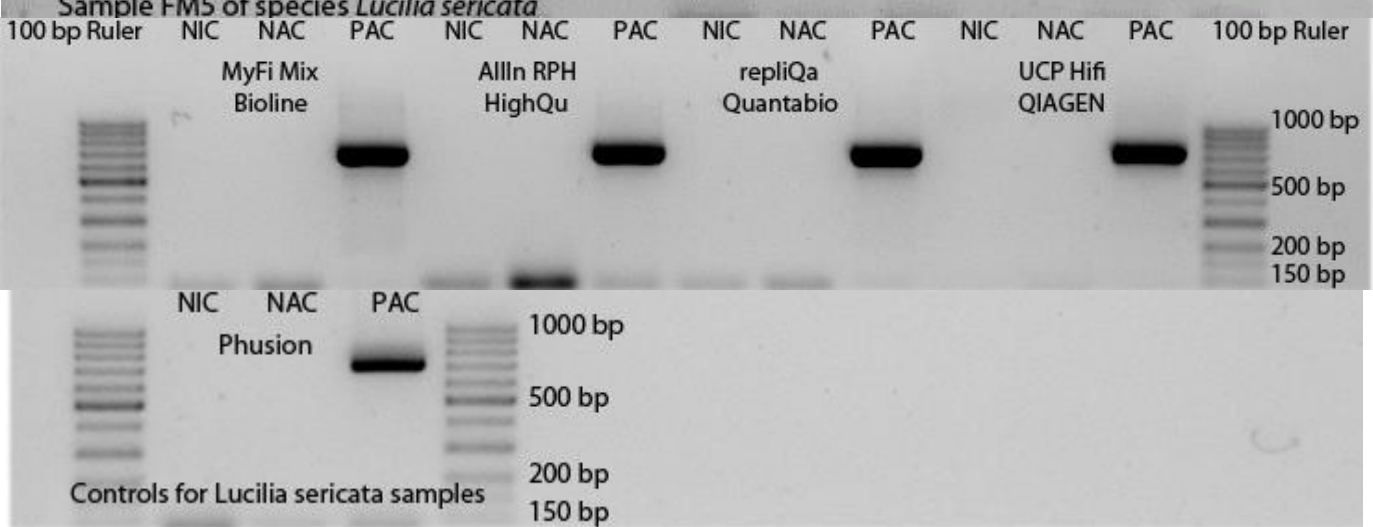

Supplement: Supplementary file 1 [file insects-13-00679-s001.zip › Figure S1.pdf]

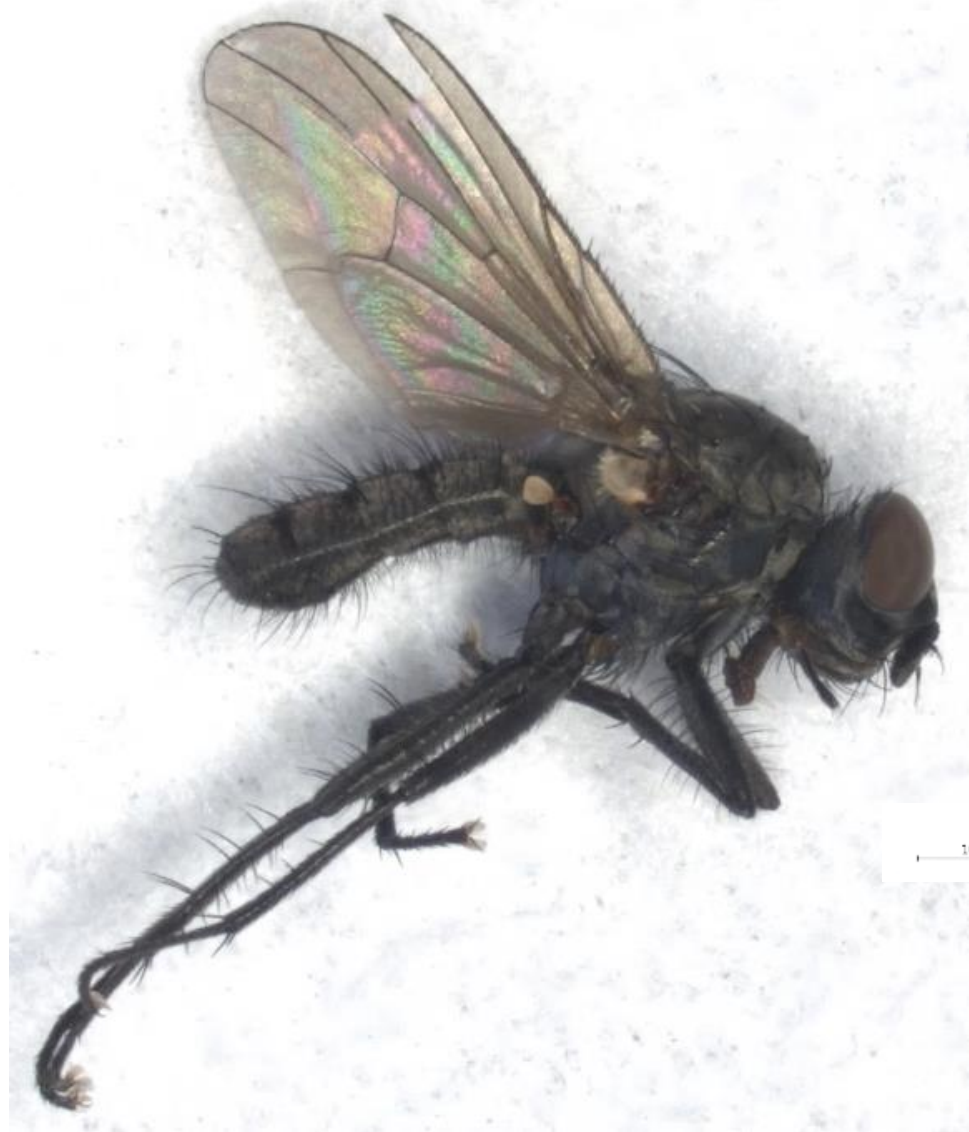

A

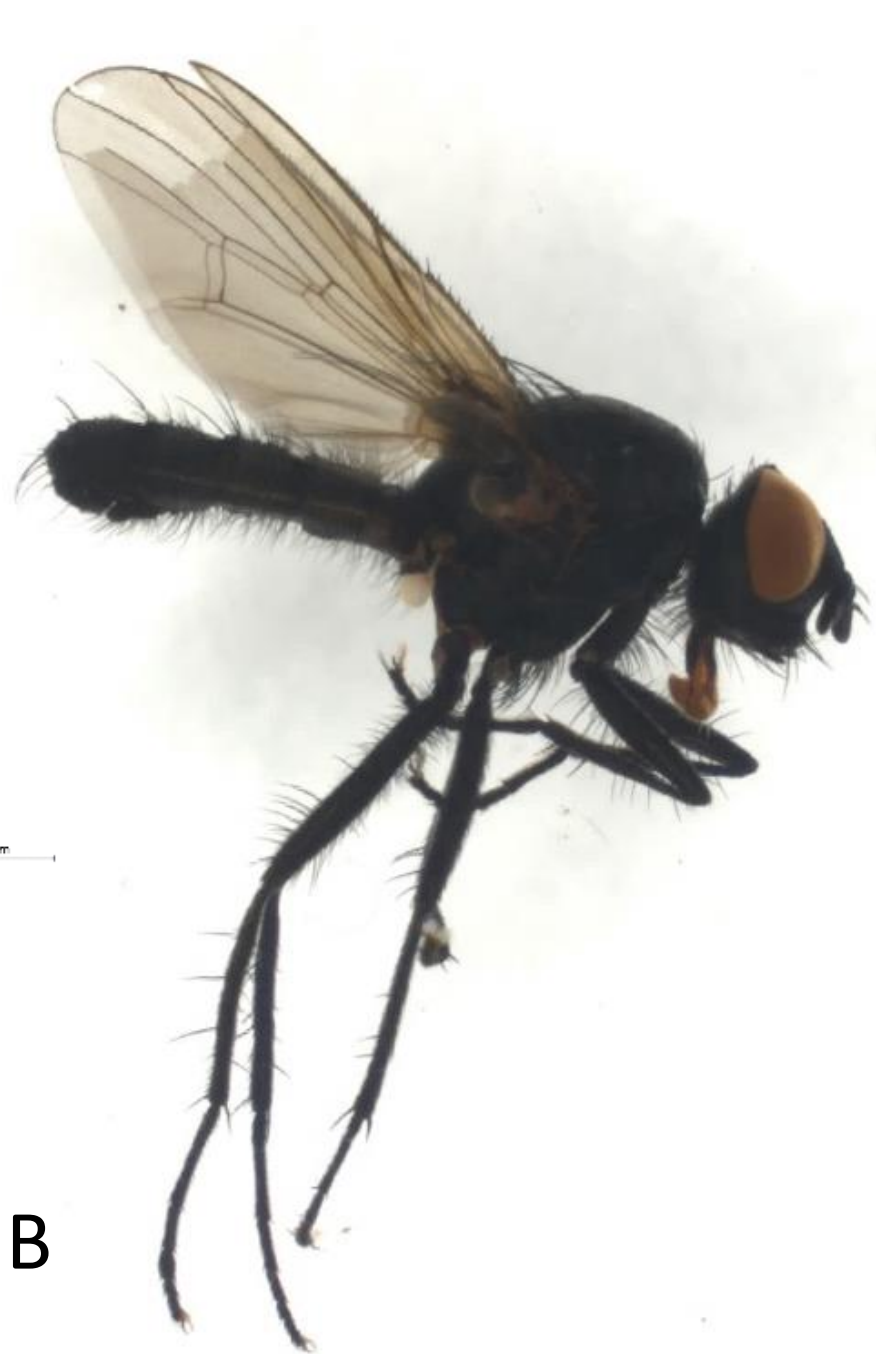

B

Supplement: Supplementary file 1 [file insects-13-00679-s001.zip › Figure S2.pdf]

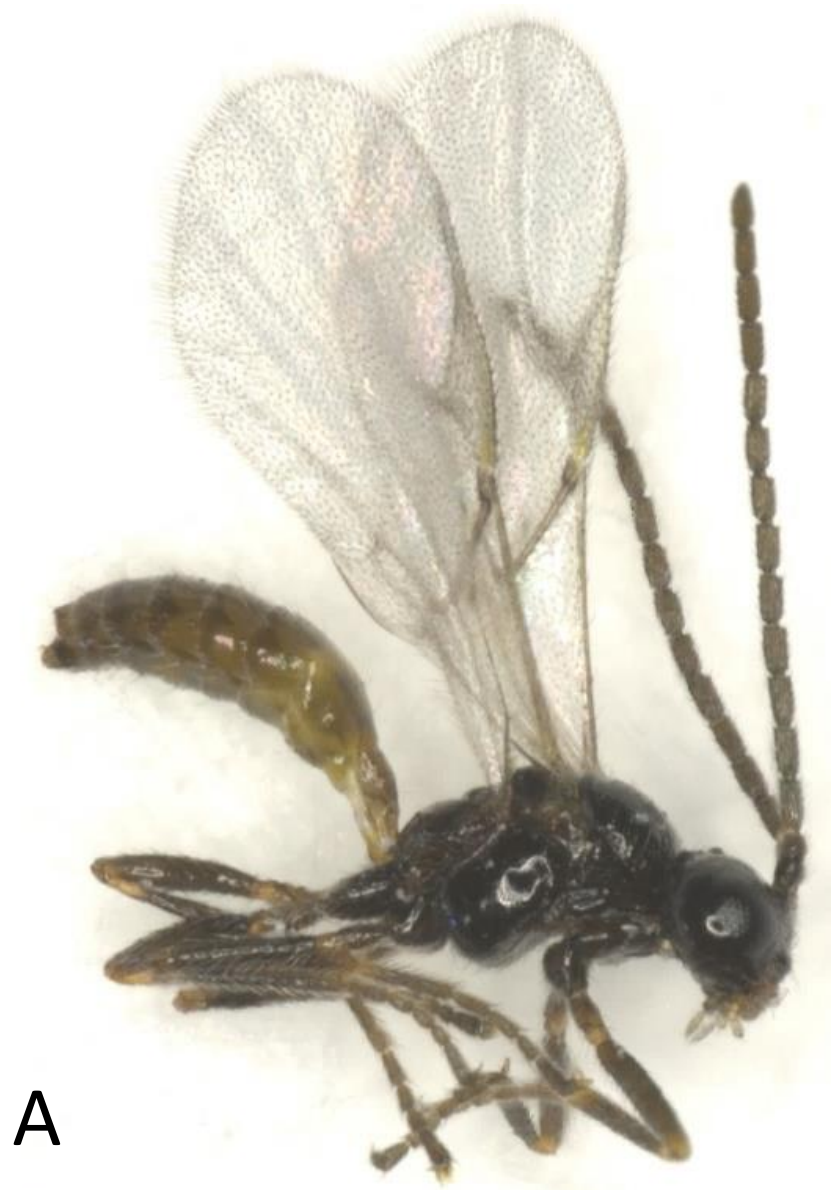

A

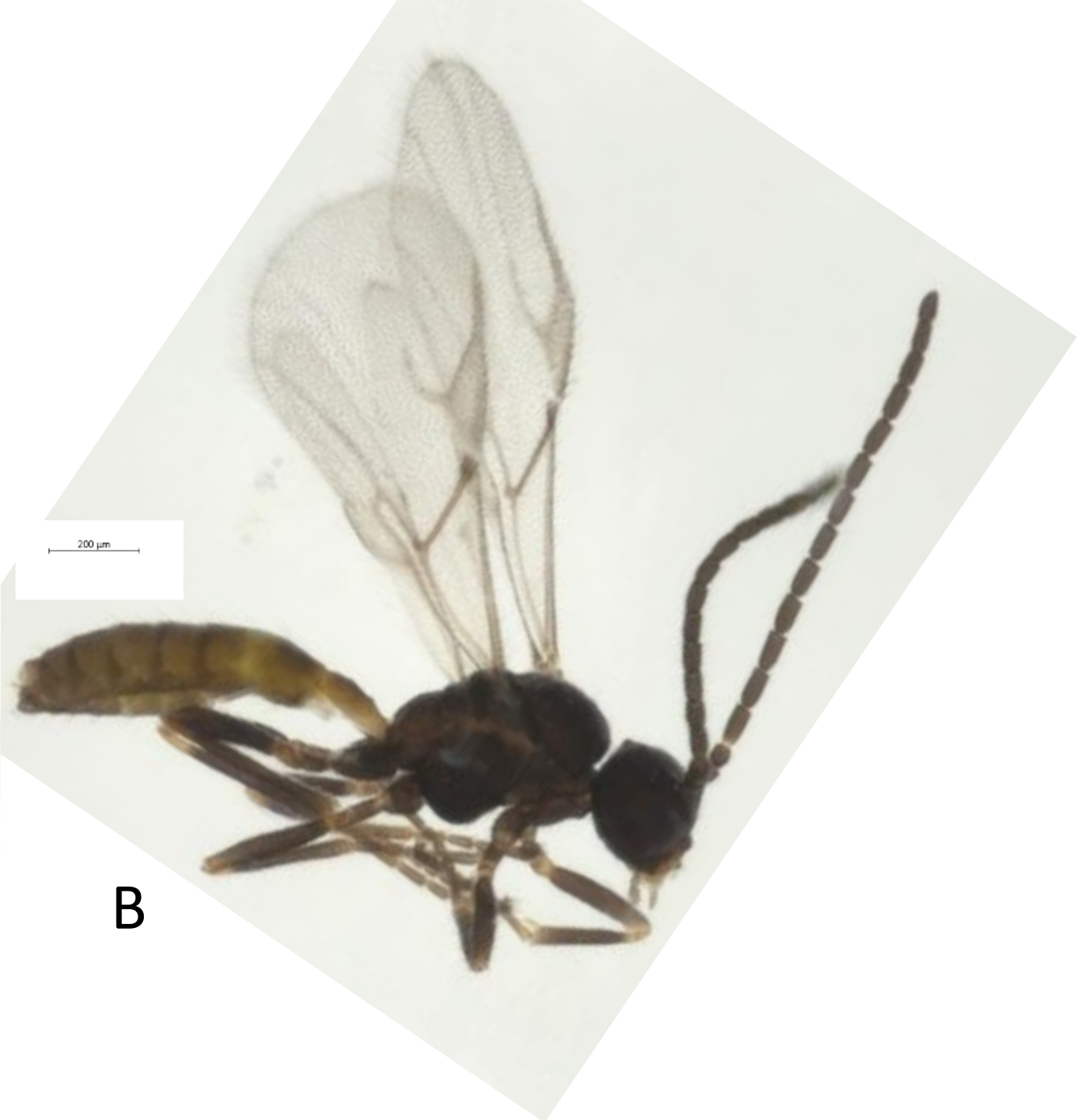

B

Supplement: Supplementary file 1 [file insects-13-00679-s001.zip › Figure S3.pdf]

A

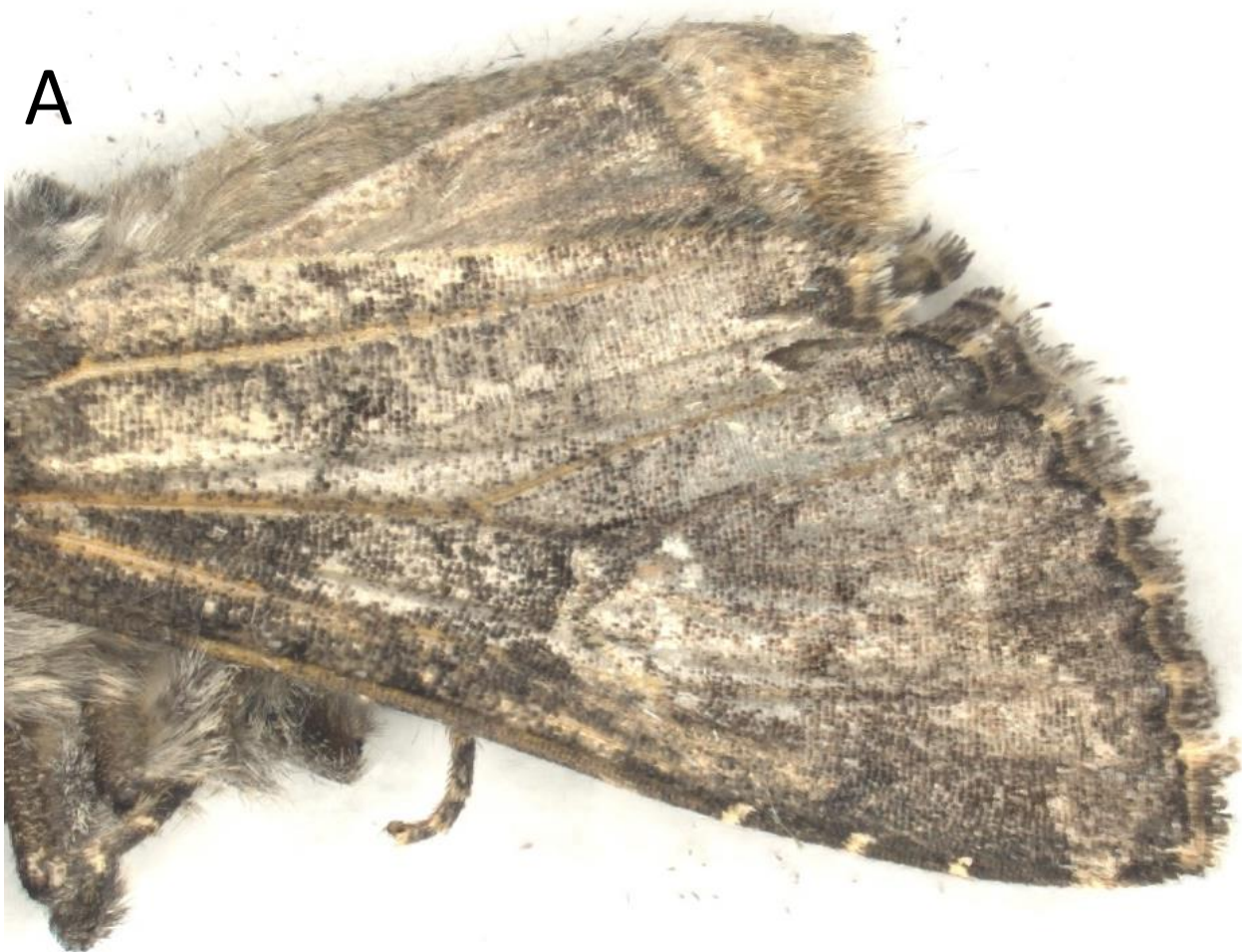

B

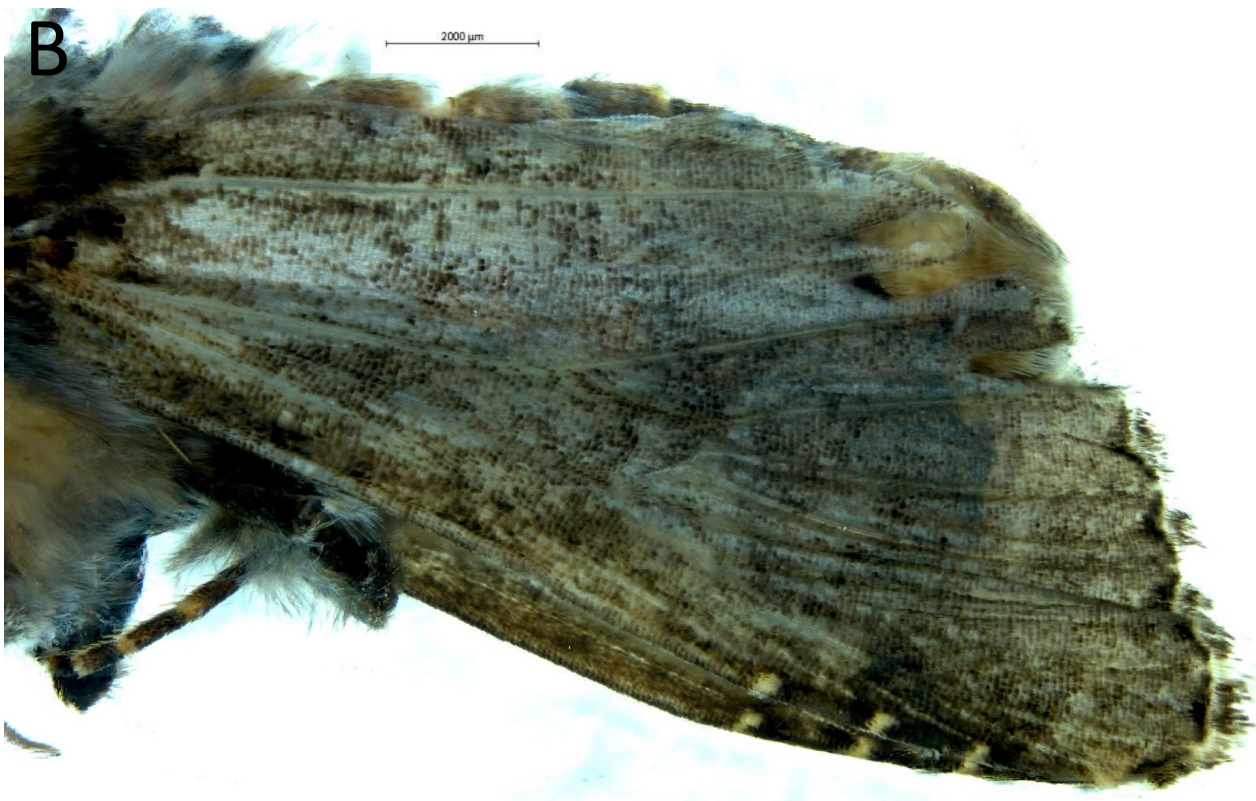

Supplement: Supplementary file 1 [file insects-13-00679-s001.zip › Figure S4.pdf]

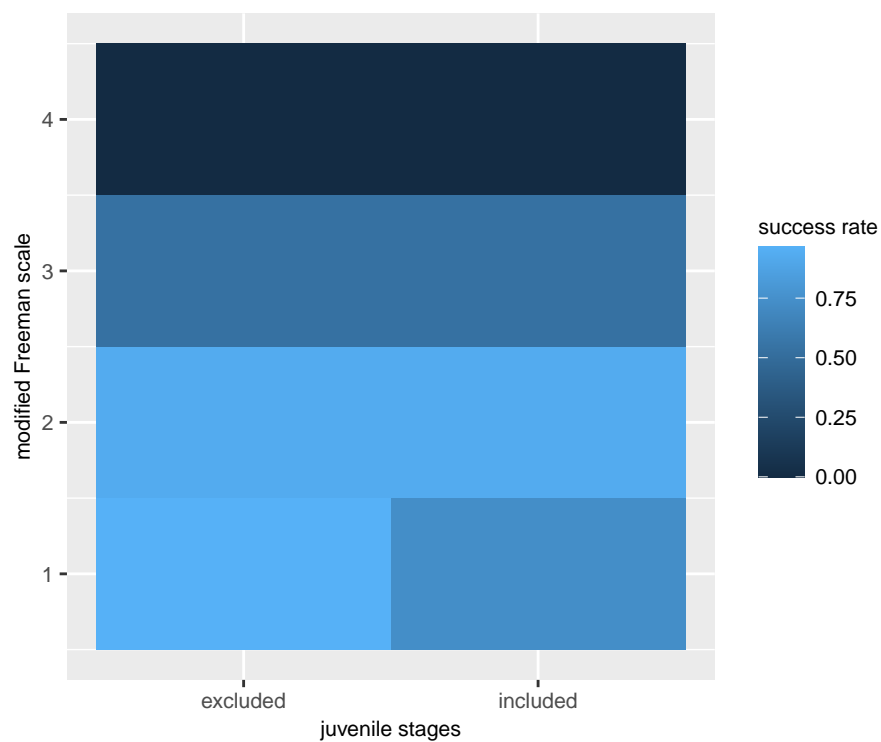

Supplement: Supplementary file 1 [file insects-13-00679-s001.zip › Figure S5.pdf]
